# Impact of left ventricular rehabilitation on surgical outcomes in patients with borderline left heart hypoplasia

**DOI:** 10.1016/j.xjon.2024.10.010

**Published:** 2024-10-18

**Authors:** Haonan Cheng, Takuya Osawa, Christoph Röhlig, Jonas Palm, Thibault Schaeffer, Carolin Niedermaier, Nicole Piber, Paul Philipp Heinisch, Christian Meierhofer, Stanimir Georgiev, Alfred Hager, Peter Ewert, Jürgen Hörer, Masamichi Ono

**Affiliations:** a Department of Congenital and Pediatric Heart Surgery, German Heart Center Munich, University Hospital of Technische Universität München, Munich, Germany; b Division of Congenital and Pediatric Heart Surgery, University Hospital of Munich, Ludwig-Maximilians-Universität, Munich, Germany; c Europäisches Kinderherzzentrum München, Munich, Germany; d Department of Pediatric Cardiology and Congenital Heart Disease, German Heart Center Munich, University Hospital of Technische Universität München, Munich, Germany; e Department of Cardiovascular Surgery, German Heart Center Munich, University Hospital of Technische Universität München, Munich, Germany

**Keywords:** biventricular repair, borderline left ventricle, left ventricle rehabilitation, hypoplastic left heart complex

## Abstract

**Objective:**

The clinical significance of left ventricular rehabilitation for borderline left ventricular hypoplasia is controversial. This study aimed to review the surgical results of patients with borderline left ventricular hypoplasia and to evaluate the impact of left ventricular rehabilitation on outcomes.

**Methods:**

Patients diagnosed with borderline left ventricular hypoplasia and surgically treated from 2018 to 2022 were included. Overall surgical outcomes were evaluated. The changes in left ventricular volumes were calculated using angiography, and age-adjusted z-score N-terminal pro-B-type natriuretic peptide levels were analyzed in patients who underwent left ventricular rehabilitation.

**Results:**

Thirty-three patients were included. Sixteen patients underwent primary biventricular repair, 3 patients underwent primary single ventricle palliation, and the remaining 14 patients underwent left ventricular rehabilitation; 9 received bilateral pulmonary artery banding and ductal stenting, 4 received central pulmonary artery banding, and 1 received ductal stenting. Of 14 patients who received left ventricular rehabilitation, 1 died, 1 underwent single ventricle palliation, 1 was waiting for further procedure, and 11 underwent biventricular repair. After biventricular repair, 2 patients died, and 1 patient developed hemodynamic failure. As a result, only 8 patients were alive and in good condition. In patients who underwent left ventricular rehabilitation, left ventricular end-diastolic volume index, end-systolic volume index, and left ventricular stroke volume index increased over time after left ventricular rehabilitation (*P* = .001, *P* = .007, and *P* = .009, respectively). The age-adjusted z-score N-terminal pro-B-type natriuretic peptide levels were stable until biventricular repair, but significantly higher in patients who presented with hemodynamic failure after biventricular repair compared with patients who did not exhibit hemodynamic failure.

**Conclusions:**

In patients with borderline left heart hypoplasia, the left ventricular rehabilitation procedure promoted an increase in left ventricular volume and contributed to establishing a biventricular circulation. The short-term results of this strategy are satisfactory, but further studies are essential to determine the long-term outcomes.

## Graphical Abstract

**Figure:**
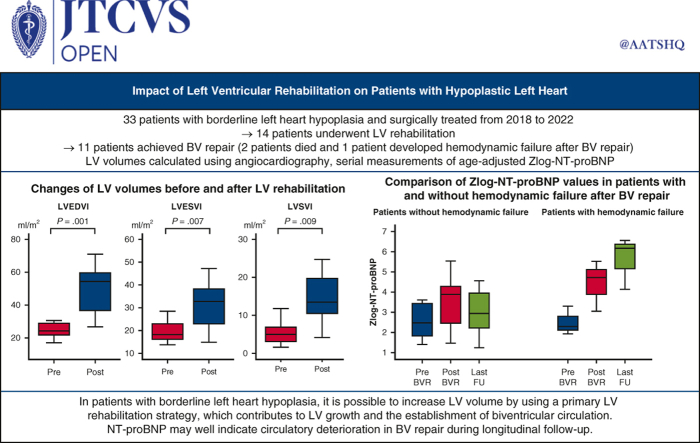

**Figure:**
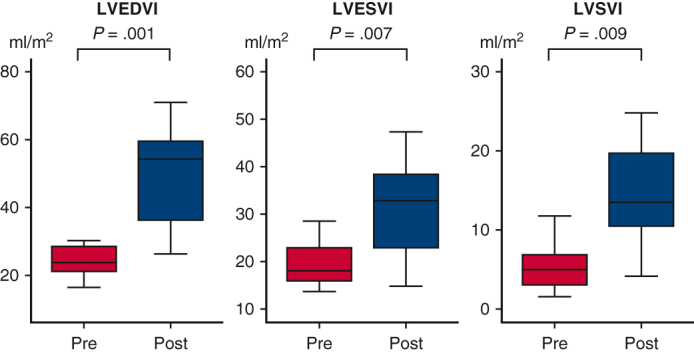
Changes of LV volume before and after LV rehabilitation. LVEDVI (*P* = .001), LVESVI (*P* = .007), and LVSVI (*P* = .009) increased after LV rehabilitation. *EDVI,* End-diastolic volume index; *ESVI,* end-systolic volume index; *LV,* left ventricle; *SVI,* stroke volume index.

**Central Message**

Patients with borderline LV hypoplasia may achieve LV growth and subsequent BV circulation after LV rehabilitation. NT-proBNP may be used to predict successful BV repairs.

**Perspective**

After LV rehabilitation, various LV volume parameters increased and even reached normal range. This LV growth allowed more patients with borderline LV hypoplasia to undergo BV repair. Zlog-NT-proBNP may indicate hemodynamic deterioration after BV repair during longitudinal follow-up; therefore, we suggest that NT-proBNP should become a routine examination in a patient's follow-up.

See Commentary Discussion on page 374.

Borderline left heart hypoplasia or hypoplastic left heart complex (HLHC) has a broad spectrum. HLHC can occur in combination with other congenital heart defects such as unbalanced atrioventricular septal defect (UAVSD) or double outlet right ventricle (DORV) and can be observed as a variant of hypoplastic left heart syndrome (HLHS). HLHC is characterized by small left-side structures including a small mitral valve (MV), a small left ventricle (LV), left ventricular outflow tract obstruction (LVOTO), a small aortic valve (AV), hypoplastic aortic arch, and aortic coarctation.^1^, ^2^, ^3^, ^4^, ^5^ The association with endocardial fibroelastosis (EFE) is frequently observed.^6^ Multilevel obstruction is described in most patients, whereas the degree of hypoplasia varies between individuals. Impaired LV systolic and diastolic function and right ventricular (RV) pressure or volume overload contribute to heart failure.^1^ Therefore, the surgical management of patients with this spectrum is always complex and highly individual.^7^, ^8^, ^9^, ^10^, ^11^ The decision toward a biventricular (BV) repair or a single ventricle (SV) palliation in newborns with HLHC is challenging because of the lack of clear consensus or criteria.

Although these children traditionally have been directed toward SV palliation, a subset of patients may be candidates for BV repair using an LV rehabilitation strategy.^12^, ^13^, ^14^, ^15^, ^16^ The decision for BV repair or SV palliation may be easier and more reliable during infancy after successful LV rehabilitation than in the neonatal period. For example, in 2009, Emani and colleagues^12^ reported on a staged BV repair using LV rehabilitation aimed to promote LV growth in patients with HLHC. In their experience, initial procedures including relief of LV inflow and outflow obstructions, resection of EFE, or restriction of the atrial septal defect (ASD) promoted satisfactory LV development. Subsequently, a few patients were able to undergo BV repair. In addition, Akintürk and colleagues^14^ demonstrated that a hybrid strategy consisting of bilateral pulmonary artery banding (PAB), ductal stenting, and the creation of a restrictive ASD increased LV volume and resulted in a higher rate of achieving BV repair. Currently, this strategy is recognized as the “LV rehabilitation strategy,” but clear indications for this strategy and reliable outcome prognosis are yet to be determined.

Since 2018, our institution adopted the LV rehabilitation strategy to a subset of patients with HLHC. The aim of this study was to review the surgical results of these patients, to evaluate the effects of LV rehabilitation on clinical outcomes, and to investigate the usefulness of N-terminal pro-B-type natriuretic peptide (NT-proBNP) as a marker of ventricular stress for the identification of successful BV repair.^17^^,^^18^

## Materials and Methods

### Data Availability Statement

The data that support the findings of this study are available from the corresponding author on reasonable request.

### Ethical Statement

The Institutional Review Board of the Technical University of Munich approved the study (No. 2023-638-S-SB on December 7, 2023) and waived the need for informed consent from the patients who were retrospectively analyzed in the study.

### Patients

All patients with borderline HLHC who had undergone at least 1 surgical procedure at the German Heart Center Munich between 2018 and 2022 were reviewed. Borderline HLHC was defined as a small non-apex–forming LV, frequently associated with a small MV, LVOTO, small AV, or hypoplastic aortic arch. Patients with an extremely small AV and MV (z-score <−5.0) were excluded as classic HLHS. Patients with complex cardiac anomalies, in whom the LV is a systemic ventricle, such as UAVSD and DORV, were included in this study. Patients with complex anatomies, in whom the LV is not considered a systemic ventricle such as atrioventricular discordance or straddling MV, were excluded from this study. Patients who underwent LV rehabilitation after the neonatal period were excluded from this study. Patients’ diagnosis, initial echocardiographic data, surgical procedures, and outcomes were evaluated. LV dimensions and hemodynamics were retrieved from preoperative and postoperative echocardiography and cardiac catheterization results.

### Echocardiography

Two-dimensional echocardiography was performed at the first admission and serially after the surgical and interventional procedures. AV diameter, MV diameter, LV end-diastolic diameter, and LV end-systolic diameter were assessed in the parasternal long-axis view. Z-scores, indicating the number of SDs the measurements deviate from the mean diameter of a normal population, were calculated as outlined by Pettersen and colleagues.^19^ LV volumes and the LV apex to RV apex ratio were measured from the apical 4-chamber view. LV volumes, including LV end-diastolic volume index (LVEDVI), LV end-systolic volume index (LVESVI), and LV stroke volume index (LVSVI), were measured from the apical 4-chamber view.^20^ On echocardiographic measurements, LVEDVI between 15 and 30 mL/m^2^ was considered an appropriate criterion for the LV rehabilitation strategy. It was also considered whether the patient had single-level or multi-level obstructions including aortic and MV hypoplasia and aortic arch hypoplasia with or without coarctation.

### Catheterization for the Measurement of Left Ventricular Structures and Hemodynamic Failure

In patients who underwent LV rehabilitation strategy, pre- and postrehabilitation LVEDVI, LVESVI, and LVSVI were calculated using serial angiocardiography and compared.^21^ Hemodynamic variables were reviewed, and hemodynamic failure after BV repair was defined as LV end-diastolic pressure more than 20 mm Hg, mean pulmonary artery pressure more than 35 mm Hg, or pulmonary vascular resistance more than 6 international Wood units, according to the report from Beattie and colleagues.^22^

### Left Ventricular Rehabilitation Procedure and Operative Technique

LV rehabilitation comprised the following surgical procedures: restriction of ASD if it is not restrictive, relief of inflow and outflow obstructive lesions (surgical aortic and mitral valvuloplasty), and EFE resection. Restriction of ASD was performed through a median sternotomy with cardiopulmonary bypass, either under cardiac arrest or ventricle fibrillation, but normally without circulatory arrest.^12^ In patients with unrestricted ASD (no pressure gradient between the left atrium and right atrium regarding the interatrial pressure gradient measured by transesophageal echocardiography), ASD was restricted using a fenestrated Gore-Tex patch (3-mm fenestration). In patients with unrestricted ASD but were considered too risky for cardiopulmonary bypass and patients with a restrictive ASD, no procedure was performed on the atrial septum. Transcatheter balloon aortic valvuloplasty combined with bilateral PAB was included in the definition of LV rehabilitation. Bilateral PAB was performed using a 3-mm polytetrafluoroethylene prosthesis in patients below 3 kg and a 3.5-mm prosthesis in patients above 3 kg of birth weight.^14^ The polytetrafluoroethylene tube was cut into 2 pieces of 3-mm length. These bands were opened, placed around the vessels, and fixed to the pulmonary adventitia with 6-0 monofilament nonabsorbable sutures. Transcatheter ductal stent (Onyx stent) implantation is performed usually 1 to 3 days after bilateral PAB.^14^

### Follow-up and Clinical Outcome

Patients were under the care of pediatric cardiologists in an outpatient setting, and most of the patients were supported by a home monitoring program.^23^ The primary end points of the study were the transplant-free survival and the achievement of BV repair. BV repair was defined as the establishment of serial systemic and pulmonary circulations without shunts, except for restrictive atrial shunts. The secondary end point was hemodynamic failure as described above. Adverse events were defined as death, transplant, takedown to SV palliation, and hemodynamic failure. NT-proBNP data were collected at various time points, including before and after LV rehabilitation, and before and after BV repair. In patients who underwent LV rehabilitation and BV repair, serial NT-proBNP values were compared using its age-adjusted z-score (zlog) because reference intervals are highly age-dependent. Calculations of zlog-NT-proBNP values were performed as previously described.^24^

### Statistical Analysis

Categorical variables were presented as absolute numbers and percentages. The chi-square test was used for categorical data, and continuous variables were expressed as medians with interquartile ranges (IQRs) or means with SD. An independent sample *t* test was used for approximately normally distributed variables, and the Mann–Whitney *U* test was applied for variables not conforming to normal distribution. Comparison of pre- and postoperative LV dimensions and zlog-NT-proBNP values were analyzed by the partially matched Wilcoxon test. Risk factor analysis for unsuccessful BV repair (death, transplantation, or hemodynamic failure after BV repair, not reaching BV repair) was performed using logistic regression model. A competitive risk analysis for SV palliation, BV repair, and death was performed. Data analysis was performed using SPSS version 28.0 for Windows (IBM) and R-statistical software (R Foundation for statistical computing).

## Results

### Patient Characteristics and Echocardiogram Data

**Figure 1:**
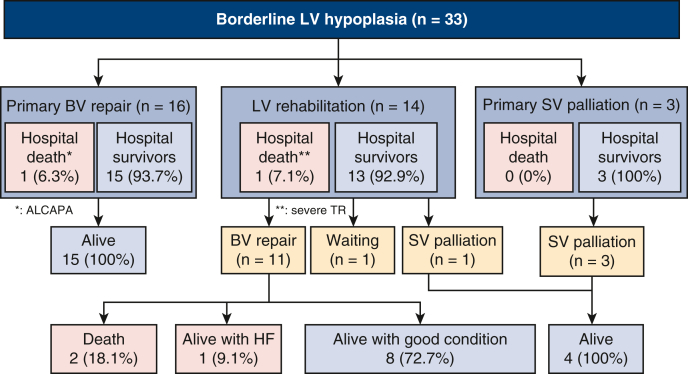
Flow chart of the patients included in this study. *LV*, Left ventricle; *BV*, biventricular; *SV*, single ventricle; *HF*, hemodynamic failure; *ALCAPA*, anomalous left coronary artery from the pulmonary artery; *TR*, tricuspid regurgitation.

A total of 33 patients with the diagnosis of borderline HLHC who were surgically treated between 2018 and 2022 were included in this study. Two patients with congenitally corrected transposition of the great arteries, 1 patient with a straddling MV, and 1 patient who underwent LV rehabilitation at 340 days were excluded from this study. Figure 1 shows a flow chart of the patient selection and their classification into the 3 treatment strategies. A total of 16 patients underwent primary BV repair, and 3 patients underwent primary SV palliation. The remaining 14 patients underwent initial LV rehabilitation. The patients’ characteristics are shown in Table 1. The main diagnosis included HLHC in 28 patients, UAVSD in 4 patients, and DORV in 1 patient. As for the left-side obstructions, a single obstructive lesion in the LV was observed in 3 patients (9.1%) and multiple obstructive lesions were observed in 30 patients (90.9%). Regarding concomitant diagnoses, EFE and restrictive ASD were observed in 7 patients (21.2%) each. Patients with LV rehabilitation had a higher incidence of intrauterine balloon dilatation of the AV (28.6% vs 0.0%, *P* = .013) and EFE (42.9% vs 5.3%, *P* = .009). The initial dimensions of the left heart structures measured on the preoperative echocardiogram are shown in Table 1.

**Table 1:** Baseline patient characteristics

Variables	Total	LV rehabilitation	Non-rehabilitation	*P* value
N (%) or median (IQR)	(n = 33)	(n = 14)	(n = 19)	
Male sex	20 (60.6)	9 (64.3)	11 (57.9)	.710
Prematurity	3 (9.1)	1 (7.1)	2 (10.5)	.738
Genetic anomalies	5 (15.2)	1 (7.1)	4 (21.1)	.271
Fetal intervention	4 (12.1)	4 (28.6)	0 (0.0)	**.013**
Primary diagnosis				
HLHC	20 (60.6)	7 (50.0)	13 (68.4)	.285
Critical AS	5 (15.2)	4 (28.6)	1 (5.3)	.065
Shone complex	3 (9.1)	2 (14.3)	1 (5.3)	.373
Hypoplastic aorta and CoA	12 (36.4)	1 (7.1)	11 (57.9)	**.003**
Borderline HLHS	8 (24.2)	4 (28.6)	4 (21.1)	.618
UAVSD	4 (12.1)	2 (14.3)	2 (10.5)	.744
DORV	1 (3.0)	1 (7.1)	0 (0.0)	.237
Associated anomalies				
EFE	7 (21.2)	6 (42.9)	1 (5.3)	**.009**
Restrictive ASD	7 (21.2)	4 (28.6)	3 (15.8)	.375
PLSVC	7 (21.2)	1 (7.1)	6 (31.6)	.090
ALCAPA	1 (3.0)	0 (0.0)	1 (5.3)	.383
Obstructive lesions				
Single	3 (9.1)	2 (14.3)	1 (5.3)	.373
Multiple	30 (90.9)	12 (85.7)	18 (94.7)	.373
Aortic arch	29 (87.9)	11 (78.6)	18 (94.7)	.156
Initial echocardiographic data				
IVS (mm)		5.8 ± 1.8	4.0 ± 1.3	**.028**
IVS Z-score		4.9 ± 1.9	2.9 ± 1.7	**.025**
LVDd (mm)		10.8 ± 2.6	10.9 ± 2.6	.939
LVDd Z-score		−5.0 ± 1.7	−4.6 ± 1.8	.665
LVDs (mm)		6.8 ± 2.9	6.4 ± 2.1	.760
LVDs Z-score		−2.6 ± 1.5	−2.6 ± 1.2	.934
LVEDVI (mL/m^2^)		14.6 ± 8.2	13.3 ± 8.3	.723
LVESVI (mL/m^2^)		5.1 ± 4.5	3.7 ± 2.3	.425
LVEF (%)		67.0 ± 24.8	70.4 ± 21.6	.851
LVa/RVa (%)		87.4 ± 14.7	91.2 ± 17.6	.759
AV diameter (mm)		5.4 ± 1.0	5.3 ± 0.8	.835
AV Z-score		−2.1 ± 1.7	−2.2 ± 1.2	.937
MV diameter (mm)		8.1 ± 1.8	8.5 ± 1.5	.593
MV Z-score		−2.1 ± 2.0	−1.6 ± 1.6	.518
Minimal arch dimension		4.2 ± 1.1	4.0 ± 1.3	.610

Bold indicates *P* < .05. *LV*, Left ventricle; *IQR*, interquartile range; *HLHS*, hypoplastic left heart syndrome; *AS*, aortic stenosis; *CoA*, coarctation of the aorta; *UAVSD*, unbalanced atrioventricular septal defect; *DORV*, double outlet right ventricle; *EFE*, endocardial fibroelastosis; *ASD*, atrial septal defect; *PLSVC*, persistent left superior vena cava; *ALCAPA*, anomalous left coronary artery from the pulmonary artery; *IVS*, interventricular septum; *LVDd*, LV end-diastolic diameter; *LVDs*, LV end-systolic diameter; *LVEDVI*, left ventricular end-diastolic volume index; *LVESVI*, left ventricular end-systolic volume index; *LVEF*, left ventricular ejection fraction; *LVa/RVa*, left ventricular apex to right ventricular apex ratio; *AV*, aortic valve; *MV*, mitral valve.

### Initial Procedure and Outcome

Sixteen patients who had an initial LVEDVI greater than 30 mL/m^2^ underwent primary BV repair. The initial procedure of these patients was performed at a median age of 8 days (IQR, 6-13). Procedures on the aorta included 14 aortic arch repairs and 2 coarctation repairs. Intracardiac procedures included 8 fenestrated ASD closures, 3 ventricular septal defect (VSD) closures, and 2 AV repairs. In 5 patients with muscular VSDs that were technically difficult to close, PAB was concomitantly performed, and these VSDs had spontaneously closed or become hemodynamic irrelevant. One patient who had a concomitant anomalous left coronary artery from the pulmonary artery died 20 days after the aortic arch repair, fenestrated ASD closure, and anomalous left coronary artery from the pulmonary artery repair (reimplantation of the left coronary artery onto the aorta) due to heart failure. The remaining 15 patients survived the procedure and were alive during a median follow-up of 3.8 years (IQR, 2.8-5.6).

In 3 patients who were not suitable for LV rehabilitation (LVEDVI <12 mL/m^2^), primary SV palliation was performed: Two patients with HLHS underwent the Norwood procedure on the 7th and 12th days of life, followed by bidirectional cavopulmonary shunt (BCPS) and total cavopulmonary connection. The other patient with UAVSD, DORV, and pulmonary stenosis had a balanced pulmonary blood flow and underwent initial BCPS at the age of 3 months. All patients undergoing primary SV palliation were alive 5.4, 6.3, and 6.8 years after BCPS.

Fourteen patients underwent LV rehabilitation. Median age and weight at the initial surgery were 7 (6-18) days and 3.3 (3.0-4.0) kg, respectively. The details of the initial procedures are provided in Table 2. The initial LV rehabilitation procedure included 9 bilateral PABs with ductal stenting, 4 central PABs, and 1 ductal stenting. Six patients had EFE, but only 1 patient underwent EFE resection. Five patients (38%) underwent ASD restriction as a concomitant procedure. One patient with borderline HLHC who had severe tricuspid regurgitation from birth died on postoperative day 22 after bilateral PAB and ductal stenting due to progressive RV failure. The remaining 13 patients survived the initial LV rehabilitation procedure and were discharged from the hospital.

**Table 2:** Details of left ventricular rehabilitation procedure

Patient	Primary Diagnosis	Obstruction					EFE	Restrictive ASD	VSD	Initial procedure (LV rehabilitation)	Age at initial procedure (d)	Hospital stay (d)	Results
		Aortic arch	CoA	AV	LVOTO	MS							
1	HLHC	1	1	1	0	1	1	0	0	AV balloon valvuloplasty, bilateral PAB, ductal stent	16	18	Alive
2	HLHC	1	0	1	0	0	1	0	0	AVP, bilateral PAB, ductal stent, fASD closure, endocardectomy	17	25	Alive
3	UAVSD	0	0	0	0	0	0	0	1	Central PAB, mVSD closure with Amplatzer	30	6	Alive
4	HLHS	1	0	1	0	1	0	0	0	Bilateral PAB, ductal stent, fASD closure	6	40	Alive
5	HLHC	1	1	1	1	1	0	0	0	Rashkind, ductal stent, CoA resection	10	N/A	Alive
6	HLHC	1	1	1	0	1	0	0	1	Central PAB, fASD closure	8	66	Alive
7	UAVSD	1	0	0	1	0	0	0	1	Central PAB	31	7	Alive
8	HLHC	0	0	1	0	1	1	1	0	Bilateral PAB, ductal stent	3	14	Alive
9	DORV	0	1	0	0	0	0	1	1	CoA resection, central PAB	18	6	Alive
10	HLHS	1	1	1	0	1	1	1	0	Bilateral PAB, ductal stent	6	19	Alive
11	HLHS	1	1	1	0	1	0	0	1	Bilateral PAB, ductal stent	4	22	Death
12	HLHS	1	1	1	0	1	0	0	1	Bilateral PAB, ductal stent, fASD closure, VSD closure	7	28	Alive
13	HLHC	1	1	1	0	1	1	0	0	Bilateral PAB, ductal stent, fASD closure	6	7	Alive
14	HLHC	0	0	1	1	1	1	1	1	Bilateral PAB, ductal stent, AV balloon valvuloplasty	5	8	Alive

*CoA*, Coarctation of the aorta; *AV*, aortic valve; *LVOTO*, left ventricular outflow tract obstruction; *MS*, mitral stenosis; *EFE*, endocardial fibroelastosis; *ASD*, atrial septal defect; *VSD*, ventricular septal defect; *LV*, left ventricle; *HLHC*, hypoplastic left heart complex; *PAB*, pulmonary artery banding; *AVP*, aortic valvuloplasty; *fASD*, fenestrated atrial septal defect; *UAVSD*, unbalanced atrioventricular septal defect; *mVSD*, muscular ventricular septal defect; *N/A*, not available; *DORV*, double outlet right ventricle.

### Biventricular Repair in Patients With Initial Left Ventricular Rehabilitation

Results of the LV rehabilitation are shown in Table 3. Among the 13 hospital survivors, 11 patients achieved BV repair after a median duration of 3.9 months (IQR, 2.0-6.6) after the initial LV rehabilitation procedure. BV repair procedures consisted of 5 Ross-Konno procedures, 4 aortic arch repairs, 2 UAVSD repairs, and 1 DORV repair. In 1 patient, the LV volume did not increase after LV rehabilitation (AV repair, bilateral PAB, fenestrated ASD closure, and endocardectomy). Instead of BV repair, this patient underwent modified Blalock–Taussig shunt, atrial septectomy, tricuspid valve repair, and AV closure 7.2 months after initial LV rehabilitation and subsequent BCPS. One remaining patient is currently waiting for BV repair. The percentage of patients who were able to achieve BV repair after LV rehabilitation was 78%.

**Table 3:** Results after left ventricular rehabilitation procedure

Patient	Diagnosis	Second procedure	Age (mo)	Repair	Results	Third procedure
1	HLHC	Ross-Konno	2.9	BVR	Death	
2	HLHC	MBTS, ASE, PA debanding, TVP, AV closure	7.8	SVP	Alive	BCPS, re-TV repair
3	UAVSD	AVSD repair, PA debanding	10.1	BVR	Alive	
4	HLHS	Aortic arch repair, PA debanding	2.1	BVR	Alive	Ross-Konno, MV repair
5	HLHC			Waiting	Alive	
6	HLHC	Re-PAB	1.3	BVR	Alive	VSD closure, PA debanding
7	UAVSD	AVSD repair	5.4	BVR	Alive	
8	HLHC	Ross-Konno	6.1	BVR	Alive	
9	DORV	ASO, VSD/ASD closure, LVOTO resection	2.6	BVR	Alive	
10	HLHS	AVP, Aortic arch repair, PA debanding	6.0	BVR	Alive with HF	Reverse Potts shunt
11	HLHS	None (death after LV rehabilitation)				
12	HLHS	AVP, Aortic arch repair, ASD/VSD closure	2.1	BVR	Alive	Ross-Konno
13	HLHC	AVP, aortic arch repair, PA debanding	4.1	BVR	Alive	
14	HLHC	Ross-Konno	2.3	BVR	Death	

*HLHC*, Hypoplastic left heart complex; *BVR*, biventricular repair; *MBTS*, modified Blalock-Taussig shunt; *ASE*, atrial septectomy; *PA*, pulmonary artery; *TVP*, tricuspid valvuloplasty; *AV*, aortic valve; *SVP*, single ventricle palliation; *BCPS*, bidirectional cavopulmonary shunt; *TV*, tricuspid valve; *UAVSD*, unbalanced atrioventricular septal defect; *AVSD*, atrioventricular septal defect; *HLHS*, hypoplastic left heart syndrome; *MV*, mitral valve; *PAB*, pulmonary artery banding; *VSD*, ventricular septal defect; *DORV*, double outlet right ventricle; *ASO*, arterial switch operation; *ASD*, atrial septal defect; *LVOTO*, left ventricular outflow tract; *AVP*, aortic valvuloplasty; *HF*, hemodynamic failure; *LV*, left ventricle.

**Figure E1:**
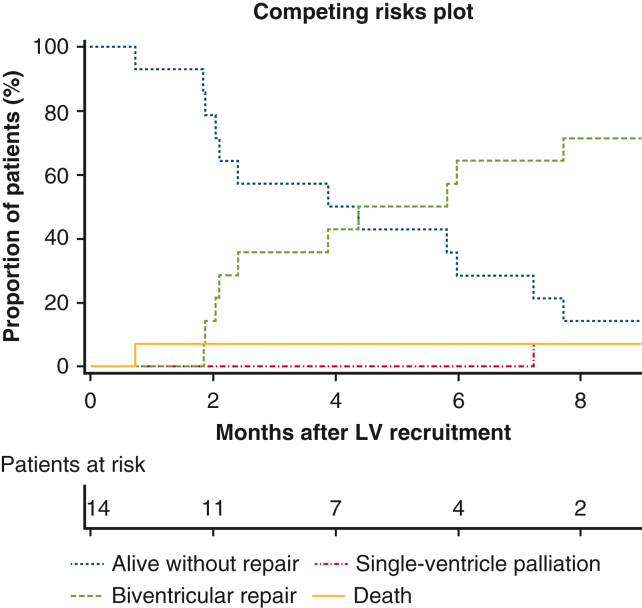
Competing risk plot showing progress in the treatment of patients who underwent LV rehabilitation - whether they were awaiting further treatment, underwent subsequent biventricular repair or single ventricle palliation, or died.

Of the 11 patients who underwent BV repair, 2 patients died on the 8th and 15th postoperative days due to heart failure and multiorgan failure, respectively. Both patients underwent the Ross–Konno procedure. The remaining 9 patients survived until a median follow-up time of 2.7 years (IQR, 2.2-4.5). Two patients with the initial diagnosis of HLHC underwent the Ross–Konno procedure 1 month and 3 years after BV repair. They were alive at the last follow-up 1 and 2 years after the procedure, respectively. A total of 4 patients required catheter intervention. Three patients underwent balloon dilatation of the pulmonary arteries. One patient developed persistent pulmonary hypertension (left ventricular end-diastolic pressure [LVEDP] of 26 mm Hg, mean pulmonary artery pressure of 55 mm Hg, and pulmonary vascular resistance of 7.2 international Woods units) and underwent reverse Potts shunt 14 months after BV repair. The outcome of this patient was interpreted as hemodynamic failure as described above. There was no transplantation or takedown to SV palliation after BV repair. A competing risks plot with the outcomes after the initial LV rehabilitation procedure is shown in Figure E1, and the corresponding 95% confidence limits are listed in Table E1.

When we compared the survival in 4 patients undergoing SV palliation and 27 patients undergoing BV repair, survival 5 years after the initial procedure was 100% and 86.2%, respectively (*P* = .445).

### Left Ventricular Growth and Hemodynamic Changes After Left Ventricular Rehabilitation

**Figure 2:**
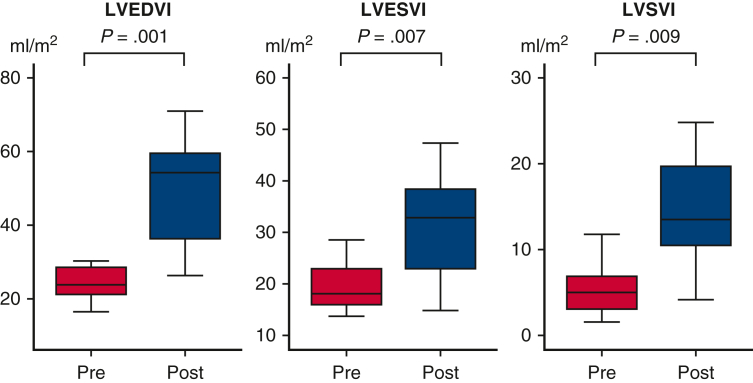
Box-and-whiskers dot plots showing serial changes of LV volume after LV rehabilitation. The upper and lower whiskers mark the minimum and maximum values, the *upper* and *lower borders* of the box represent the upper and lower quartiles, and the *middle horizontal line* represents the median. *LVEDVI*, Left ventricular end-diastolic volume index; *LVESVI*, left ventricular end-systolic volume index; *LVSVI*, left ventricular stroke volume index.

LV volumes before and after LV rehabilitation were measured using angiography (n = 11). After LV rehabilitation, in patients who achieved BV repair, LVEDVI increased during the interval between LV rehabilitation and BV repair (25.1 ± 6.5 to 54.2 ± 25.5 mL/m^2^, *P* = .001) between median ages of 16 (6-126) days and 86 (59-230) days (Figure 2). LVESVI (19.8 ± 4.6 to 35.1 ± 18.9 mL/m^2^, *P* = .007) and LVSVI (5.3 ± 3.0 to 19.2 ± 14.3 mL/m^2^, *P* = .009) also increased after LV rehabilitation. In the 1 patient who underwent SV palliation, LVEDVI had not reached 30 mL/m^2^ after LV rehabilitation (21.7 to 26.3 mL/m^2^). Hemodynamic changes before and after LV rehabilitation are shown in Table E2. Although left atrial pressure, left ventricular pressure, and aortic pressure increased, LVEDP decreased with LV rehabilitation. The left heart dimensions before and after LV rehabilitation are shown in Table E3. By echocardiographic evaluation, LV apex to RV apex ratio increased from 87.8% ± 14.3% to 100.9% ± 15.6% after LV rehabilitation (*P* = .05).

### Zlog N-Terminal Pro-B-type Natriuretic Peptide Levels From Before Left Ventricular Rehabilitation to After Biventricular Repair

**Figure 3:**
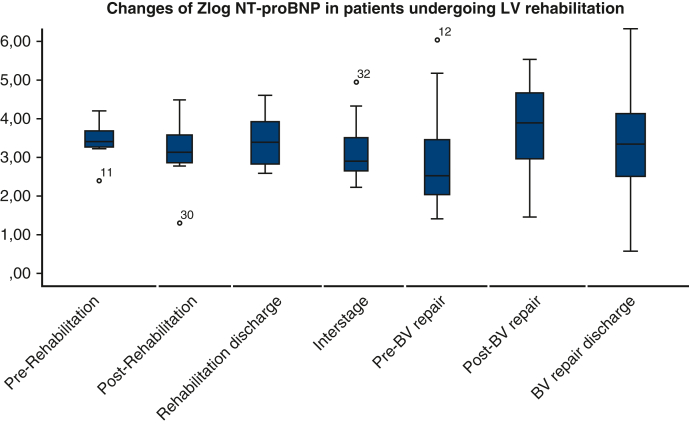
Box-and-whiskers dot plots showing serial changes of zlog value of NT-proBNP. The upper and lower whiskers mark the minimum and maximum values, the *upper* and *lower borders* of the box represent the upper and lower quartiles, and the *middle horizontal line* represents the median. *NT-proBNP*, N-terminal pro-B-type natriuretic peptide; *LV*, left ventricle; *BV*, biventricular.

**Figure 4:**
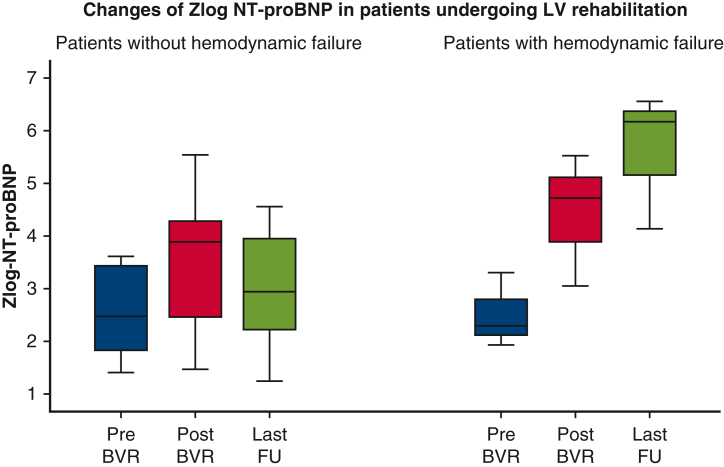
Box-and-whiskers dot plots comparing zlog value of NT-proBNP in patients with and without hemodynamic failure after BV repair. The upper and lower whiskers mark the minimum and maximum values, the *upper* and *lower borders* of the box represent the upper and lower quartiles, and the *middle horizontal line* represents the median. *NT-proBNP*, N-terminal pro-B-type natriuretic peptide; *BV*, biventricular; *LV*, left ventricle; *BVR*, biventricular repair; *FU,* follow-up.

Serial zlog-NT-proBNP measurements were conducted on 13 patients, with a total of 77 measurements before and after LV rehabilitation and BV repair (Figure 3). Zlog-NT-proBNP levels slowly declined from prerehabilitation to pre-BV repair. Because the variance of levels after BV repair is considerable compared with preoperatively, we further evaluated the association between zlog-NT-proBNP and clinical status (Figure 4). The 3 patients who died or had hemodynamic failure after BV repair had significantly higher zlog-NT-proBNP levels than those who did not.

### Risk Factor Analysis for Unsuccessful Biventricular Repair

Among 14 patients who underwent LV rehabilitation, risk factor analysis for unsuccessful BV repair is shown in Table E4. The association of EFE (odds ratio [OR], 14.00, *P* = .055), LV end-systolic diameter z-score (OR, 2.253, *P* = .089), and preoperative LV ejection fraction (OR, 0.938, *P* = .060) had a *P* value less than .1 in the univariable analysis. Among patients who underwent LV rehabilitation and subsequent BV repair, 2 patients with EFE and 1 patient without EFE experienced failing BV repair.

## Discussion

**Figure 5:**
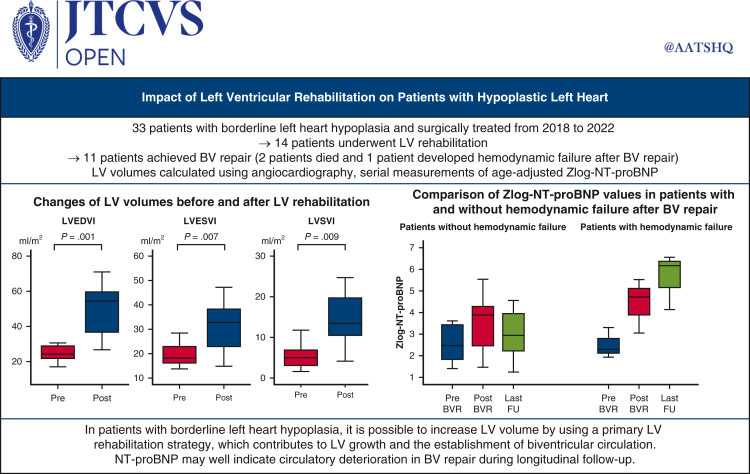
Brief summary of this article. *LV*, Left ventricle; *BV*, biventricular; *NT-proBNP*, N-terminal pro-B-type natriuretic peptide; *LVEDVI*, LV end-diastolic volume index; *LVESVI*, left ventricular end-systolic volume index; *LVSVI*, left ventricular stroke volume index; *BVR*, biventricular repair; *FU,* follow-up.

This retrospective study reports our experience with the surgical management of HLHC. LV rehabilitation was performed in 42% of the patients, and 78% of them were able to survive BV repair. Early mortality after LV rehabilitation was caused by associated severe tricuspid regurgitation. BV repair was unsuccessful in 2 patients who died of heart failure after the Ross-Konno procedure. LV volume was significantly increased in 11 patients who could eventually reach BV repair. The LV rehabilitation procedure was associated with low operative mortality and acceptable survival after BV repair (Figure 5 and Video 1).

### Diagnosis and Surgical Strategy of Borderline Left Ventricle

The surgical management of patients with hypoplastic LV requires careful patient selection.^1^, ^2^, ^3^, ^4^, ^5^ Although definitive cases are directed toward staged SV palliation, transplantation, or BV repair, the decision becomes intricate in cases with borderline hypoplasia of the LV. Traditionally, LV hypoplasia unsuitable for BV repair is defined by an LVEDVI of less than 20 mL/m^2^.^25^ However, the measurement of LV volume in the patient with borderline LV is often difficult. Grosse-Wortmann and colleagues^26^ reported that echocardiography consistently underestimated LV volume and did not correlate with magnetic resonance imaging.^26^ In older patients, magnetic resonance imaging is the established gold standard for ventricular volumetry, yet in newborns or infants with congenital heart disease, no such standard has been established because of technical difficulties. Therefore, we usually calculated LV volume using angiography. In this study, patients who underwent LV rehabilitation presented with a mean LVEDVI of 25 mL/m^2^, and the minimal LVEDVI was 12 mL/m^2^ in angiography. As for the differences among LV rehabilitation, primary BV repair, and SV palliation, patients who underwent LV rehabilitation had a thicker interventricular septum than those who underwent primary BV repair (shown in Table E5). The interventricular septum is related to ventricular diastolic dysfunction and EFE.

### Candidacy for Biventricular Repair

A previous study reported that an LVEDVI of 20 mL/m^2^ may be a cutoff value for successful BV repair.^25^ The presence of EFE and the function of the rehabilitated LV also may determine the outcome. Elevated LVEDP and pulmonary hypertension were found to elevate the risk at BV repair.^27^ Most of the patients in this series underwent LV rehabilitation in the neonatal period. The LV volume increased in almost all patients within several months after LV rehabilitation, with the exception of only 1 patient, whose LV did not grow after LV rehabilitation. Other studies with older patients demonstrated worse rates of BV repair.^25^^,^^27^ We assume that early rehabilitation may have better effects than a late intervention. Catheter-based neonatal interventions incorporated into an LV rehabilitation strategy play an important role in the relief of LVOTO, thereby allowing an optimal timing of BV repair.

### Timing of and Results After Biventricular Repair

Sojak and colleagues^16^ reported on BV repair after a hybrid palliation in 27 patients at a median age of 83 days (range, 15-371). Twenty-three patients (85.2%) were alive at a median of 3.3 years after BV repair. They suggested that a hybrid palliation can stimulate LV growth and that more patients may eventually achieve BV circulation than initially thought. The Giessen group performed BV repair at a median age of 156 days (range, 51-525).^14^^,^^15^ In their opinion, the optimal time point to decide on BV repair or definitive palliation after the hybrid approach is approximately 5 months of age. We observed similar experiences. The median age at BV repair was 4.1 months. It seems reasonable to accomplish the BV repair not too late, as long as the criteria for successful BV repair are presented. On the other hand, a short rehabilitation period may contribute to postoperative LV failure. Both patients who died after LV rehabilitation underwent the Ross–Konno procedure for BV repair at 3 months of age. It may be better to perform such a highly invasive procedure after more rehabilitation time. Furthermore, prolonged LV diastolic dysfunction may develop and result in elevated RV pressures after BV repair. In this study, 1 patient even needed a Potts shunt to decrease supra-systemic RV pressures and to increase systemic circulation. In another patient, elevated left atrial pressure was treated by creating an ASD with a stent. Further studies are mandatory to clarify the long-term results of LV rehabilitation strategy after BV repair. Because of the complex nature of HLHC, the reintervention rate after BV repair was reported as high.^28^ In this study, 2 patients needed the Ross–Konno procedure after BV repair. Four patients required catheter interventions, most of which were pulmonary artery related.

### Left Ventricular Growth and Risk Factors After Left Ventricular Rehabilitation

It is clear that ventricular size matters, and LVEDV is frequently used to determine size adequacy. The Giessen group was the first to report that neonatal hybrid palliation increased median LVEDVI (mL/m^2^).^14^^,^^15^ On the other hand, Emani and colleagues demonstrated that a more aggressive rehabilitation including restriction of ASD, resection of EFE, and aortic or mitral valvuloplasty promoted the development of the LV and improved LV function.^12^, ^13^ Both strategies are recognized as LV rehabilitation strategy. The Giessen approach can postpone the decision whether BV or SV repair is suitable without complicated procedures. However, this approach does not relieve LVOTO in the setting of an obstructive aortic arch, which may limit LV growth or lead to increased LVEDP. This may be one of the reasons for poor outcomes after BV repair. Moreover, this approach is disadvantaged by its stent-related complications, and there might be less LV growth after the Giessen approach than after other aggressive rehabilitations. Nevertheless, our study based on the Giessen approach demonstrated that LV end-diastolic/end-systolic volume index and LV stroke volume index increased over time after LV rehabilitation. Likewise, LV sizes were borderline in our patients in the neonatal period, whereas they reached normal sizes after LV rehabilitation. The existence of a restrictive ASD (3-3.5 mm size) is crucial for LV growth. In patients with UAVSD, LVEDVI of greater than 30 mL/m^2^ may be adequate, whereas in patients with HLHC, 35 mL/m^2^ is a safer cutoff.^29^^,^^30^ However, LV volume is not the only determinant and other parameters are relevant. For example, Shimada and colleagues^31^ demonstrated that the interventricular septum thickness was negatively associated with BV repair.

The management of EFE is also an important issue. In patients with EFE, ventricular noncompliance compounds ventricular hypoplasia. Lofland and colleagues^3^ demonstrated that the degree of EFE is a risk factor for mortality after BV repair, and Tuo and colleagues^5^ reported that the presence of EFE with consequent diastolic dysfunction is more important than LV volume in determining the outcome. In this study, among the 5 patients diagnosed with EFE, 4 reached BV repair, and 1 with severe EFE underwent SV palliation despite endocardectomy concomitant with LV rehabilitation. Two of 4 patients had adverse events after BV repair. Thus, patients with EFE should be selected cautiously for BV repair indication, and we should consider performing EFE resection more aggressively. Furthermore, we should carefully consider the timing of BV repair after rehabilitation. Yet, EFE resection during the neonatal or early infant period often can be inadequate and cause complications. Therefore, we believe it is important to perform EFE resection as many times as possible until the LV is sufficiently well. In this study, the median age of BV repair after LV rehabilitation was 4 months of age, and we think this timing of BV repair was too young to perform effective EFE resection. Therefore, we reconsider that BV repair should be performed later in patients with EFE until effective EFE resection is possible. As for the strategy, we prefer to keep the hybrid arrangement with a patent ductus arteriosus stent and loosen pulmonary artery banding until the patient is old enough for BV repair and EFE resection.

The bottom-line size does matter, but it is not enough just to have an adequate size, and the interactions between various components of the left heart are complex and difficult to predict with traditional scoring algorithms. Perhaps in the future, technologies such as artificial intelligence and computer simulation may improve sensitivity and specificity. A lingering controversy is well captured by the quip “a good SV is better than 2 bad ventricles.” Patient selection for BV repair deserves careful consideration and depends on an individual center's comfort level with the surgical, interventional, and postoperative care elements necessary for BV repair. Patients with risk factors for the Fontan such as pulmonary vein stenosis, RV dysfunction, atrioventricular valve regurgitation, single lung physiology, or high pulmonary vascular resistance have a poor long-term prognosis with SV palliation. In such patients, BV repair is easily justified. On the other hand, in patients without any risk factors, outcomes after the Fontan procedure can be excellent for many years.^32^ Nevertheless, long-term prospective studies including quality of life are needed to compare outcomes in these 2 populations.

### N-Terminal Pro-B-Type Natriuretic Peptide and Its Zlog Value as Biomarkers for Left Ventricular Rehabilitation

In this study, zlog-NT-proBNP indicated that the LV seems to gradually adapt to the “training” from the initial LV rehabilitation to the corrective operation. In addition, our results demonstrated that zlog-NT-proBNP increased after the onset of failing BV repair. Although individual values and specific cutoffs could not be identified because of a limited case load within this study, zlog-NT-proBNP is a useful parameter for suboptimal BV hemodynamics and may well indicate circulatory deterioration in BV repair during longitudinal follow-up. Because reference intervals of NT-proBNP significantly change during infancy and thus at the time of LV rehabilitation, the use of age-adjusted zlog-NT-proBNP instead of absolute values seems mandatory.

### Study Limitations

This study was limited by its retrospective, nonrandomized, single-center design. Although measurements of echocardiograms were taken several times, a small discrepancy can make a big difference, especially in infants. Furthermore, LV volume was measured by catheter angiography and echocardiograms, which makes it difficult to compare data with the other reports, in which cardiac magnetic resonance imaging is the typical modality used for calculation of ventricular volumes. Because of the small number of patients, the results should be interpreted cautiously. In addition, the definitions of borderline hypoplastic LV and rehabilitation were different in each previous report and may not be consistent with our study. This study included different diagnoses and a small number with HLHS, which is a limitation. Assignment to the therapy was not regulated by a fixed protocol but was the result of individual discussion in a board consisting of pediatric cardiovascular surgeons and pediatric cardiologists.

## Conclusions

In patients with borderline LV and small left-sided structures, it is possible to increase LV volume by using a primary LV rehabilitation strategy, which contributes to LV growth and the establishment of a BV circulation. The major advantage of this strategy is that the timing of the decision between BV repair and SV palliation can be postponed until infancy (∼5 months of age). The short-term results of this strategy are satisfactory, and further studies in larger cohorts are essential to determine the long-term outcomes.

### Webcast

You can watch a Webcast of this AATS meeting presentation by going to: https://www.aats.org/resources/impact-of-left-ventricular-rec-7101.

**Figure:**
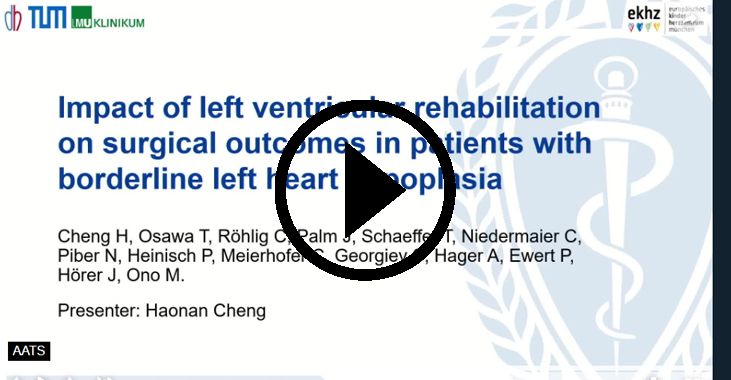

## Conflict of Interest Statement

The authors reported no conflicts of interest.

The *Journal* policy requires editors and reviewers to disclose conflicts of interest and to decline handling or reviewing manuscripts for which they may have a conflict of interest. The editors and reviewers of this article have no conflicts of interest.

## Figures and Tables

**Table E1:** The 95% confidence interval of Figure E1

Months after LV rehabilitation	Patients at risk	Alive CI 5%	Alive CI 95%	SV CI 5%	SV CI 95%	BV CI 5%	BV CI 95%	Death CI 5%	Death CI 95%
1	14	0.803	1.000					0.011	0.472
2	13	0.692	1.000			0.011	0.472	0.011	0.472
3	12	0.598	1.000			0.040	0.515	0.011	0.472
4	11	0.513	0.995			0.079	0.584	0.011	0.472
5	10	0.435	0.950			0.125	0.654	0.011	0.472
6	9	0.363	0.899			0.177	0.721	0.011	0.472
7	8	0.296	0.844			0.234	0.785	0.011	0.472
8	7	0.234	0.785			0.296	0.844	0.011	0.472
9	6	0.177	0.721			0.363	0.899	0.011	0.472
10	5	0.125	0.654			0.435	0.950	0.011	0.472
11	4	0.079	0.584	0.011	0.472	0.435	0.950	0.011	0.472
12	3	0.040	0.515	0.011	0.472	0.513	0.995	0.011	0.472
13	2	0.011	0.472	0.011	0.472	0.598	1.000	0.011	0.472
14	1	0.011	0.472	0.011	0.472	0.598	1.000	0.011	0.472

*LV*, Left ventricle; *SV*, single ventricle; *BV*, biventricular.

**Table E2:** Hemodynamic variables before and after left ventricle rehabilitation

N = 11	Prerehabilitation	Postrehabilitation	*P* value
Cardiac catheterization			
RAP	8 (6-10)	8 (5-10)	.226
RVP	73 (66-86)	77 (73-90)	.315
RVEDP	10 (10-11)	11 (10-14)	.351
PAP	44 (25-45)	44 (37-53)	.431
LAP	11 (8-16)	12 (10-14)	**.018**
LVP	88 (66-118)	95 (73-111)	**.021**
LVEDP	15 (11-17)	14 (12-18)	**.002**
AoP systolic	67 (61-88)	76 (60-90)	**.031**
AoP diastolic	34 (30-42)	37 (29-48)	.202
AoP mean	47 (43-53)	51 (40-64)	.709
SO2	82 (77-92)	83 (72-88)	.598

Bold indicates *P* < .05. *RAP*, Right atrial pressure; *RVP*, right ventricular pressure; *RVEDP*, right ventricular end-diastolic pressure; *PAP*, pulmonary artery pressure; *LAP*, left atrial pressure; *LVP*, left ventricular pressure; *LVEDP*, left ventricular end-diastolic pressure; *AoP*, aortic pressure; *SO2*, oxygen saturation.

**Table E3:** Echocardiographic measurements before and after left ventricular rehabilitation

Variables	Prerehabilitation	Postrehabilitation	*P* value
IVS (mm)	5.5 (4.0-6.0)	6.0 (4.5-6.2)	.652
IVS Z-score	4.8 (3.2-5.4)	4.0 (2.6-4.9)	.136
LVDd (mm)	10.0 (8.8-12.3)	16.0 (12.5-20.0)	**.030**
LVDd Z-score	−5.4 (−6.3-4.2)	−1.9 (−5.2 to −1.0)	.057
LVDs (mm)	5.0 (4.3-8.8)	10.0 (6.8-12.0)	**.013**
LVDs Z-score	−3.5 (−3.9-1.4)	−1.9 (−25.2 to −1.0)	.261
LVEDVI (mL/m^2^)	10.7 (8.2-18.9)	34.1 (13.2-40.0)	**.013**
LVESVI (mL/m^2^)	3.3 (1.0-7.3)	7.2 (2.5-9.8)	.237
LVEF (%)	79 (49-89)	78 (61-88)	.779
LVa/RVa (%)	84.1 (75.8-98.1)	102.1 (93.3-111.4)	**.050**
AV diameter (mm)	6.0 (4.8-6.0)	7.0 (6.0-8.0)	**<.001**
AV Z-score	−1.4 (−2.6-1.0)	−1.2 (−2.6 to −0.3)	.426
MV diameter (mm)	8.0 (7.0-9.3)	12.0 (11.0-14.0)	**<.001**
MV Z-score	−1.8 (−3.4-0.15)	0.52 (−1.7-1.9)	**.003**

Bold indicates *P* < .05. *IVS*, Interventricular septum; *LVDd*, LV end-diastolic diameter; *LVDs*, LV end-systolic diameter; *LVEDVI*, left ventricular end-diastolic volume index; *LVESVI*, left ventricular end-systolic volume index; *LVEF*, left ventricular ejection fraction; *LVa/RVa*, left ventricular apex to right ventricular apex ratio; *AV*, aortic valve; *MV*, mitral valve.

**Table E4:** Risk factor analysis for predictors of the unsuccessful biventricular repair

Variables	OR	Univariate	*P* value
		95% CI	
Age at LV rehabilitation	0.919	0.786-1.075	.293
Fetal intervention	0.600	0.041-8.732	.708
Birth weight	1.000	0.997-1.004	.839
Multiple LVOTO	0.833	0.090-7.675	.872
Critical aortic stenosis	7.000	0.501-97.75	.148
EFE	14.00	0.944-207.60	**.055**
Initial catheterization			
LAP	1.580	0.834-2.993	.161
LVEDP	1.976	0.720-5.422	.186
PAP	1.033	0.841-1.268	.760
LVEDVI	0.796	0.564-1.123	.194
LVESVI	0.757	0.496-1.154	.195
LVSVI	0.794	0.480-1.312	.368
Initial echocardiography			
LVDd Z-score	1.280	0.629-2.608	.496
LVDs Z-score	2.253	0.883-5.749	**.089**
LVEDVI	1.021	0.887-1.175	.772
LVESVI	1.284	0.931-1.770	.128
LVEF	0.938	0.877-1.003	**.060**
MV Z-score	0.848	0.470-1.530	.584
AV Z-score	0.753	0.376-1.509	.423
IVSd	1.420	0.679-2.969	.352
IVSd Z-score	1.305	0.673-2.530	.431

Bold indicates *P* < .1. *LV*, Left ventricle; *LVOTO*, left ventricular outflow tract; *EFE*, endocardial fibroelastosis; *LAP*, left atrial pressure; *LVEDP*, left ventricular end-diastolic pressure; *PAP*, pulmonary artery pressure; *LVEDVI*, left ventricular end-diastolic volume index; *LVESVI*, left ventricular end-systolic volume index; *LVSVI*, left ventricular stroke volume index; *LVDd*, LV end-diastolic diameter; *LVDs*, LV end-systolic diameter; *LVEF*, left ventricular ejection fraction; *MV*, mitral valve; *AV*, aortic valve; *IVSd*, end-diastolic interventricular septum.

**Table E5:** Echocardiographic data

Variables	LV rehabilitation	Primary BVR	SV palliation	*P* value
N (%) or median (IQR)	(n = 14)	(n = 16)	(n = 3)	
IVS (mm)	5.8 ± 1.8	3.3 ± 0.5	6.0 ± 0	**.012**
IVS Z-score	4.9 ± 1.9	2.1 ± 0.1	5.5 ± 0.1	**.004**
LVDd (mm)	10.8 ± 2.6	11.7 ± 2.6	8.5 ± 0.7	.320
LVDd Z-score	−5.0 ± 1.7	−4.1 ± 1.8	−6.2 ± 0.8	.305
LVDs (mm)	6.8 ± 2.9	7.0 ± 1.4	4.5 ± 3.5	.500
LVDs Z-score	−2.6 ± 1.5	−2.3 ± 0.7	−3.6 ± 2.1	.543
LVEDVI (mL/m^2^)	14.6 ± 8.2	15.0 ± 8.7	7.2 ± 0.5	.471
LVESVI (mL/m^2^)	5.1 ± 4.5	4.2 ± 2.1	2.2 ± 3.0	.605
LVEF (%)	67.0 ± 24.8	74.0 ± 34.0	69.1 ± 20.4	.927
LVa/RVa (%)	87.4 ± 14.7	100.0 ± 7.8	65.2 ± 7.5	**.012**
AV diameter (mm)	5.4 ± 1.0	5.3 ± 0.9	5.0 ± 0	.929
AV Z-score	−2.1 ± 1.7	−2.2 ± 1.2	−2.4 ± 0	.987
MV diameter (mm)	8.1 ± 1.8	8.7 ± 1.4	7.5 ± 2.1	.599
MV Z-score	−2.1 ± 2.0	−2.2 ± 2.1	−1.4 ± 1.6	.697

Bold indicates *P* < .05. *LV*, Left ventricle; *BVR*, biventricular repair; *SV*, single ventricle; *IQR*, interquartile range; *IVS*, interventricular septum; *LVDd,* LV end-diastolic diameter; *LVDs*, LV end-systolic diameter; *LVEDVI*, left ventricular end-diastolic volume index; *LVESVI*, left ventricular end-systolic volume index; *LVEF*, left ventricular ejection fraction; *LVa/RVa*, left ventricular apex to right ventricular apex ratio; *AV*, aortic valve; *MV*, mitral valve.

## Supplementary Data

Video 1 The author Haonan Cheng briefly explains the importance and relevance of the study. Video available at: https://www.jtcvs.org/article/S2666-2736(24)00352-8/fulltext.
