# Supplementary figures and images for: Impact of left ventricular rehabilitation on surgical outcomes in patients with borderline left heart hypoplasia
Source: JTCVS Open. 2024 Oct 18;24:359–73. doi: 10.1016/j.xjon.2024.10.010 (PMC12039426; doi:10.1016/j.xjon.2024.10.010)

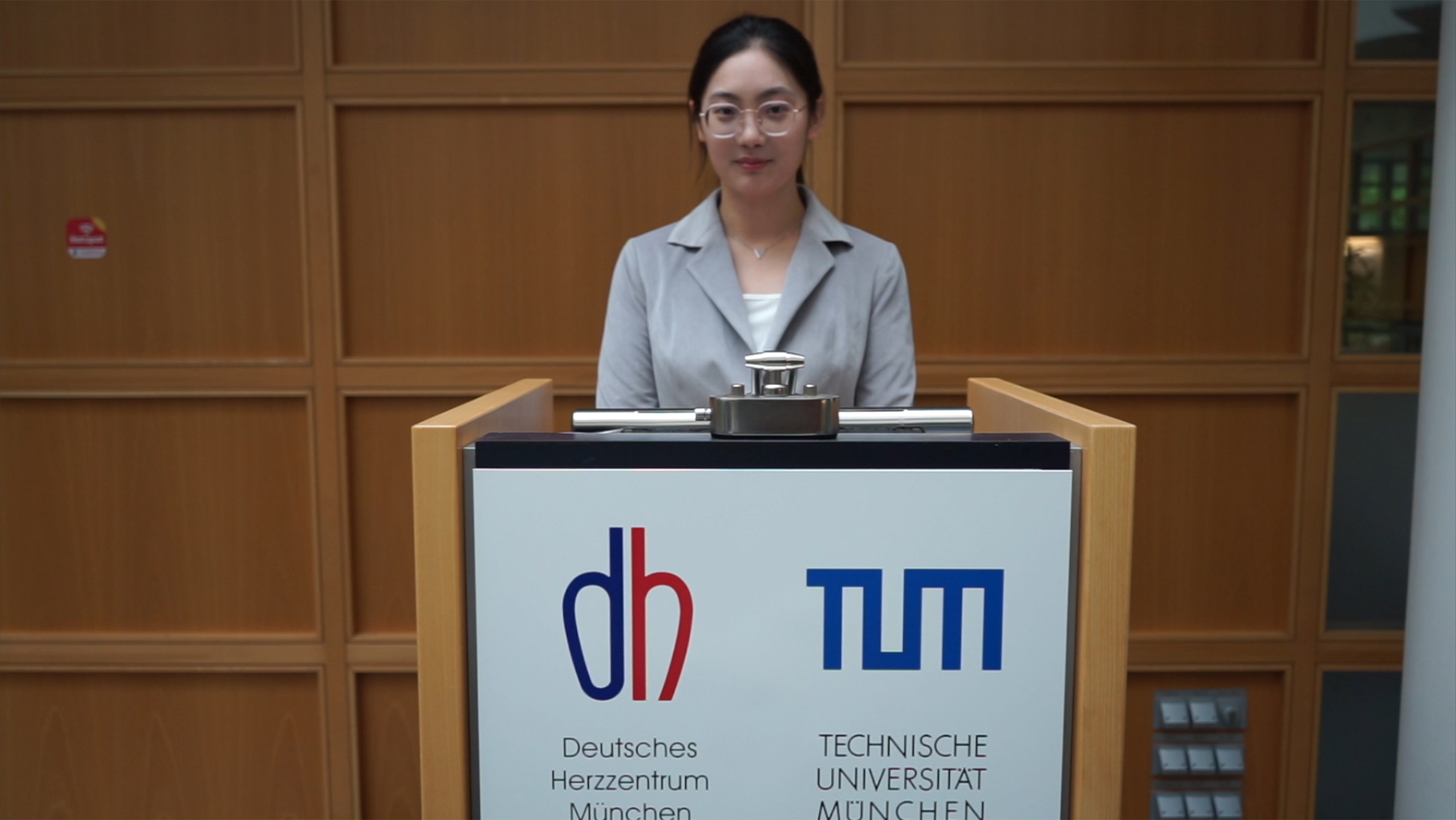

Supplement: Video 1 — The author Haonan Cheng briefly explains the importance and relevance of the study. Video available at: https://www.jtcvs.org/article/S2666-2736(24)00352-8/fulltext. [file fx3.jpg]
